# Socio-demographic patterns in hospital admissions and accident and emergency attendances among young people using linkage to NHS Hospital Episode Statistics: results from the Avon Longitudinal Study of Parents and Children

**DOI:** 10.1186/s12913-019-3922-7

**Published:** 2019-02-26

**Authors:** Leigh Johnson, Rosie Cornish, Andy Boyd, John Macleod

**Affiliations:** Population Health Sciences, Bristol Medical School, University of Bristol, Oakfield House, Oakfield Grove, Bristol, BS8 2BN UK

**Keywords:** Socio-economic status, Children and young people, Record linkage, Hospital admissions, Accident and emergency attendance, NHS, ALSPAC

## Abstract

**Background:**

In England emergency hospital admissions among children are increasing and the under 25s are the most frequent attenders of A&E departments. Children of lower socio-economic status (SES) have poorer health outcomes and higher hospital admission rates. NHS Hospital Episode Statistics (HES) are increasingly being used for research but lack detailed data on individual characteristics such as SES. We report the results of an Avon Longitudinal Study of Parents and Children (ALSPAC) study that linked the data of 3,189 consenting participants to HES. We describe rates of hospital admission, emergency readmissions, and A&E attendances and examine socio-demographic correlates of these.

**Methods:**

Subjects were singletons and twins enrolled in ALSPAC who had provided consent for linkage to their health records by the study cut-off date (31.02.12). Linkage was carried out by the Health and Social Care Information Centre (now NHS Digital). We examined rates of admissions between birth and age 20 and A&E attendances between 14 and 20 years. Socio-demographic information collected in ALSPAC questionnaires during pregnancy were used to examine factors associated with admissions, emergency readmissions (an emergency admission within 30 days of discharge) and A&E attendances.

**Results:**

Excluding birth records, we found at least one admission for 1,792/3,189 (56.2%) participants and 4,305 admissions in total. Admission rates were highest in the first year of life. Among males, admissions declined until about age 5 and then remained relatively stable; conversely, among females, they increased sharply from the age of 15. ICD 10 chapters for diseases of the digestive system and injury and poisoning accounted for the largest proportions of admissions (15.8 and 14.5%, respectively). Tooth decay was the highest single cause of admission for those aged 5–9 years. Overall, 1,518/3,189 (47.6%) of participants attended A&E at least once, with a total of 3,613 attendances between age 14 and 20 years. Individuals from more deprived backgrounds had higher rates of admissions, readmissions and A&E attendances.

**Conclusions:**

Linkage between cohort studies such as ALSPAC and HES data provides unique opportunities for detailed insights into socio-demographic and other determinants of hospital activity, which can inform secondary care demand management in the NHS.

**Electronic supplementary material:**

The online version of this article (10.1186/s12913-019-3922-7) contains supplementary material, which is available to authorized users.

## Background

Rates of accident and emergency (A&E) attendance and hospital admissions to NHS hospitals are unprecedented. Admissions via A&E departments have risen from 2.5 million in 2003/4 to 4.1 million in 2015/16, contributing to rising service costs that have led to serious concerns about the financial and operational sustainability of the NHS [1]. While older people have more emergency admissions, children and young people (under 25) are more frequent users of A&E services than the over 25s [2] and emergency admissions for infants, children and young people are increasing. There were 14% more emergency admissions of children and young people to NHS hospitals 2015/16 than in 2006/7 [3]. Children of lower socio-economic status (SES) have poorer health outcomes than children of higher SES [4–7], and higher rates of hospital admission and A&E attendances have been seen for lower SES children [8–12, 31]. Higher rates of emergency readmission (an emergency admission within 30 days of the last) have also been seen for lower SES patients [13, 14]. Descriptions of SES in health outcomes are often made using proxy neighbourhood area measures such as the English indices of deprivation [15, 16] but, due to misclassification bias, area measures have been shown to have weaker associations with health outcomes compared to individual level measures [17].

Routine health data such as those held within the NHS Hospital Episode Statistics (HES) are an invaluable resource for – amongst others – service evaluation, informing health service planning, and monitoring population-level trends in outcomes. They are also being increasingly used to answer a wide range of other types of research question and have great potential in this respect. However, one of the disadvantages of routine health data is that they lack detailed information on individual characteristics. Linkage between routine health datasets and longitudinal birth cohort studies, which have collected detailed data on individual level characteristics across the life course, provides a unique opportunity to explore determinants of objectively-measured outcomes – including health service use. Further, such outcomes will be recorded for all individuals, regardless of their participation in research studies, thus overcoming problems of attrition and non-response. Despite this, within England, ethico-legal barriers to data access and concerns regarding participant acceptability have meant few cohorts are currently able to exploit these opportunities [18].

We report results on 3,189 participants from the Avon Longitudinal Study of Parents and Children (ALSPAC) who consented to health data linkage by the Health and Social Care Information Centre (HSCIC, now NHS Digital) to NHS Hospital Episode Statistics (HES). We describe rates of hospital admissions and emergency readmissions among these participants up to age 20, and describe rates of A&E attendance using an extract of A&E records from the start of routine, centralised A&E data collation in 2007 when ALSPAC participants were aged between 14 and 16. Using a range of individual level measures collected by ALSPAC questionnaires, we examine whether socio-demographic factors are associated with hospital admissions and A&E attendances.

## Methods

### The Avon Longitudinal Study of Parents and Children (ALSPAC)

ALSPAC is a transgenerational prospective birth cohort study investigating influences on health and development across the life course. The study has been described in detail before [19]. In summary, 14,541 pregnant women who were resident in and around Bristol, England, and due to deliver between April 1, 1991 and December 31, 1992 were initially recruited, resulting in 14,062 live births and 13,988 children who were alive at one year. Subsequent phases of recruitment increased the number of enrolled children to 14,701 (15,247 pregnancies), 14,664 of whom were singletons and twins and have not subsequently withdrawn from the study. The children and their families have been followed up intensively through questionnaires, study clinics and through linkage to routine datasets. Further information about ALSPAC is given on the study website [20]. There is also a searchable dictionary of available data [21].

### Linkage to Hospital Episode Statistics (HES)

Linkage of the ALSPAC data with HES was enabled by a previous exercise which linked ALSPAC participants to the NHS Central Register. This was carried out by the HSCIC with a 99% match rate [19]. The participants were linked on NHS number, name, date of birth, sex and postcode (including postcode change over time). Linkage was conducted using automated deterministic linkage algorithms, with manual operator match of participants not linked via the automated process.

**Fig. 1:**
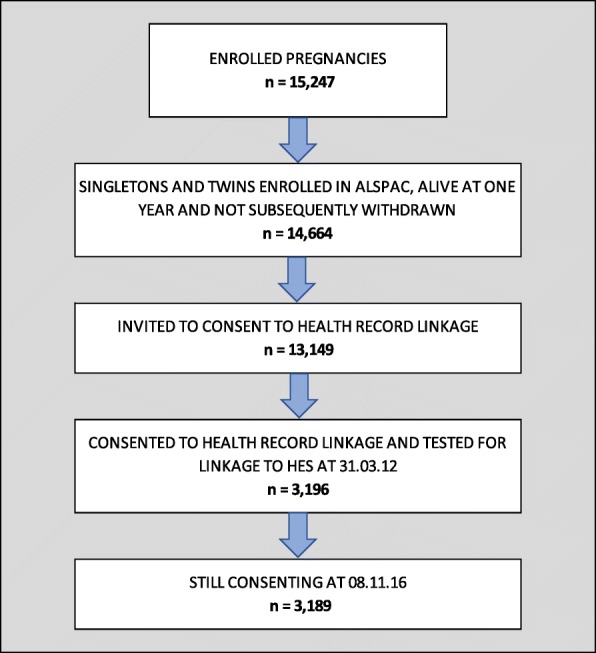
The ALSPAC enrolment campaign flow diagram

The Project to Enhance ALSPAC through Record Linkage (PEARL) involved a postal ‘fair processing’ campaign to inform participants about the extraction and use of their health and other administrative records when participants were re-enrolled at 18 years of age. An information pack and consent/objection form were sent to 13,149 enrolled singletons and twins. By 31 December 2012, (the cut-off date at which consenters were selected for inclusion into an initial linkage to the HES data set), 3,196 (24.3%) had consented to linkage to their health records. Of the 3,196 consenters, 3,189 were still consenting at the time of this analysis (Fig. 1). PEARL obtained ethical approval from the ALSPAC Ethics and Law Committee and NHS Research Ethics Committee Haydock Ref: 9/H1010/70 (protocol number 1278).

### Hospital Episode Statistics (HES)

The HES database (Copyright© 2012, re-used with the permission of the Health and Social Care Information Centre. All rights reserved) contains the records of all hospital admissions, outpatient appointments and A&E attendances at NHS hospitals in England. Some admissions in HES may occur in non-NHS clinical settings (if NHS-funded), and private patients treated in NHS hospitals are included [22, 23]. Admitted Patient Care data has been routinely collected since 1989/90, covering the life course of the ALSPAC participants. The ALSPAC linkage team received linked A&E data from the HSCIC for the first five financial years that it had been collected (from 2007/8 to 2011/12). The age range of the 3,189 study participants was 14–20 years during this period.

### The ALSPAC measures

A number of socio-demographic variables were derived from questionnaires administered during pregnancy and early infancy: maternal age, marital status (married/separated, divorced, widowed or never married), parity (the number of previous pregnancies resulting in either a livebirth or a stillbirth), ethnicity and highest education level (degree or A level/O level, CSE or vocational); and family occupational social class, which was based on the higher of the mother’s or partner’s occupational social class and dichotomised into non-manual (I to IIINM: professional, managerial or skilled non-manual occupations) and manual (IIIM to V: skilled manual, partly skilled or unskilled occupations).

### Data analysis

The main descriptive analysis included all 3,189 individuals. The analysis of socio-demographic correlates of hospital admissions, A&E attendances and readmissions was restricted to the 2,774 (87%) individuals with complete covariate information. Our analysis of readmissions includes emergency admissions occurring within 30 days of discharge from hospital [24–26]. To take account of over-dispersion, we used negative binomial regression to model rates of admissions, readmissions and attendances. We used restricted cubic splines [27] with 5 knots to adjust for age. All analyses were carried out in Stata versions 14 and 15 [28].

## Results

### Hospital admissions (Admitted Patient Care (APC) data)

Our linked data contained a total of 7,154 admissions for 3,023 (94.8%) of the 3,189 ALSPAC participants who consented to health linkage*.* There were no admitted patient care records for 166 (5.2%) participants. Of these admissions 2,849 (39.8%) were birth records. We have excluded these admissions for the purposes of these analyses and included the remaining 4,305 admissions. Of the 3,189 participants 1,397 (43.8%) had no admissions, giving a mean admission rate of 1.3 per participant (median 1, range 0–54). Table 1 gives the frequency and overall number of admissions for males and females. Altogether, 1,792/3,189 (56.2%) of participants had at least one admission, and 47 (1.5%) had nine or more. A slightly greater proportion of females than males had no admissions (44.9% compared to 42.2%). The proportions with between one and eight admissions were similar among males and females but the proportion of females with nine or more admissions was double that among males (1.8% vs. 0.9%). The mean admission rate was 1.3 (median 1, range 0–15) in males and 1.4 (median 1, range 0–54) in females. Among the females, six individuals accounted for a total of 200 (7.7%) of the 2,612 admissions.

**Table 1:** Frequency and total number of hospital admissions, A&E attendances and emergency readmissions by sex

	Frequency	Male (*n* = 1,281)	Female (*n* = 1,908)	Overall (*n* = 3,189)
Admissions	0	540 (42.2%)	857 (44.9%)	1,397 (43.8%)
	1	347 (27.1%)	502 (26.3%)	849 (26.6%)
	2	174 (13.6%)	263 (13.8%)	437 (13.7%)
	3	91 (7.1%)	107 (5.6%)	198 (6.2%)
	4	53 (4.1%)	63 (3.3%)	116 (3.6%)
	5	30 (2.3%)	40 (2.1%)	70 (2.2%)
	6	16 (1.2%)	17 (0.9%)	33 (1.0%)
	7	7 (0.5%)	9 (0.5%)	16 (0.5%)
	8	11 (0.9%)	15 (0.8%)	36 (1.1%)
	9+	12 (0.9%)	35 (1.8%)	47 (1.5%)
Total number of admissions		1,693	2,612	4,305
Emergency readmissions	0	1,241 (96.9%)	1,788 (93.7%)	3,029 (95.0%)
	1	35 (2.7%)	77 (4.0%)	112 (3.5%)
	2+	5 (0.4%)	43 (2.3%)	48 (1.5%)
Total number of readmissions		46	268	314
A&E attendances	0	642 (50.1%)	1,029 (53.9%)	1,671 (52.4%)
	1	294 (23.0%)	437 (22.9%)	731 (22.9%)
	2	165 (12.9%)	212 (11.1%)	377 (11.8%)
	3	63 (4.9%)	92 (4.8%)	155 (4.9%)
	4	37 (2.9%)	57 (3.0%)	94 (2.9%)
	5	39 (3.0%)	24 (1.3%)	63 (2.0%)
	6	14 (1.1%)	16 (0.8%)	30 (0.9%)
	7	9 (0.7%)	15 (0.8%)	24 (0.8%)
	8	8 (0.6%)	6 (0.3%)	14 (0.4%)
	9+	10 (0.8%)	20 (1.0%)	30 (0.9%)
Total number of attendances		1,481	2,132	3,613

**Fig. 2:**
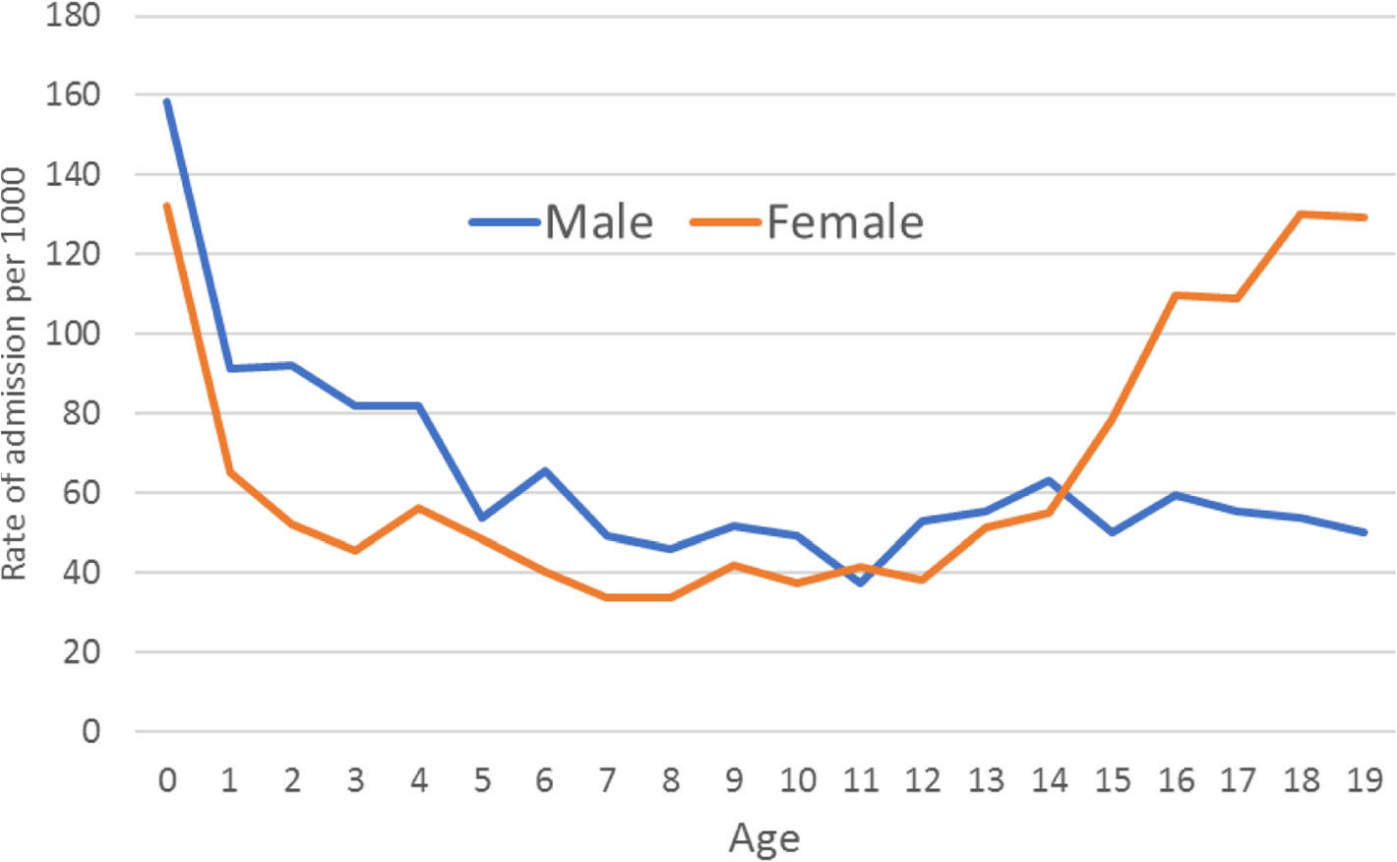
Rates of hospital admission by age and sex (males: *n* = 1,693 admissions, 1,281 individuals) (females: *n* = 2,612 admissions, 1,908 individuals)

Among both males and females, rates of admission were highest in the first year of life, gradually declined to age 7 years, and remained relatively stable until age 11. Thereafter, the age distribution was markedly different in males compared to females. Among males, the rate of admission remained reasonably stable, whereas admission rates among females sharply increased from the age of 15. (Fig. 2). After excluding the six individuals who accounted for a relatively large proportion of the admissions, this pattern remained - although was slightly attenuated (Additional file 1: Figure S1).

### Primary diagnoses

Of the 4,305 admissions, all but one had a primary diagnosis recorded.

**Fig. 3:**
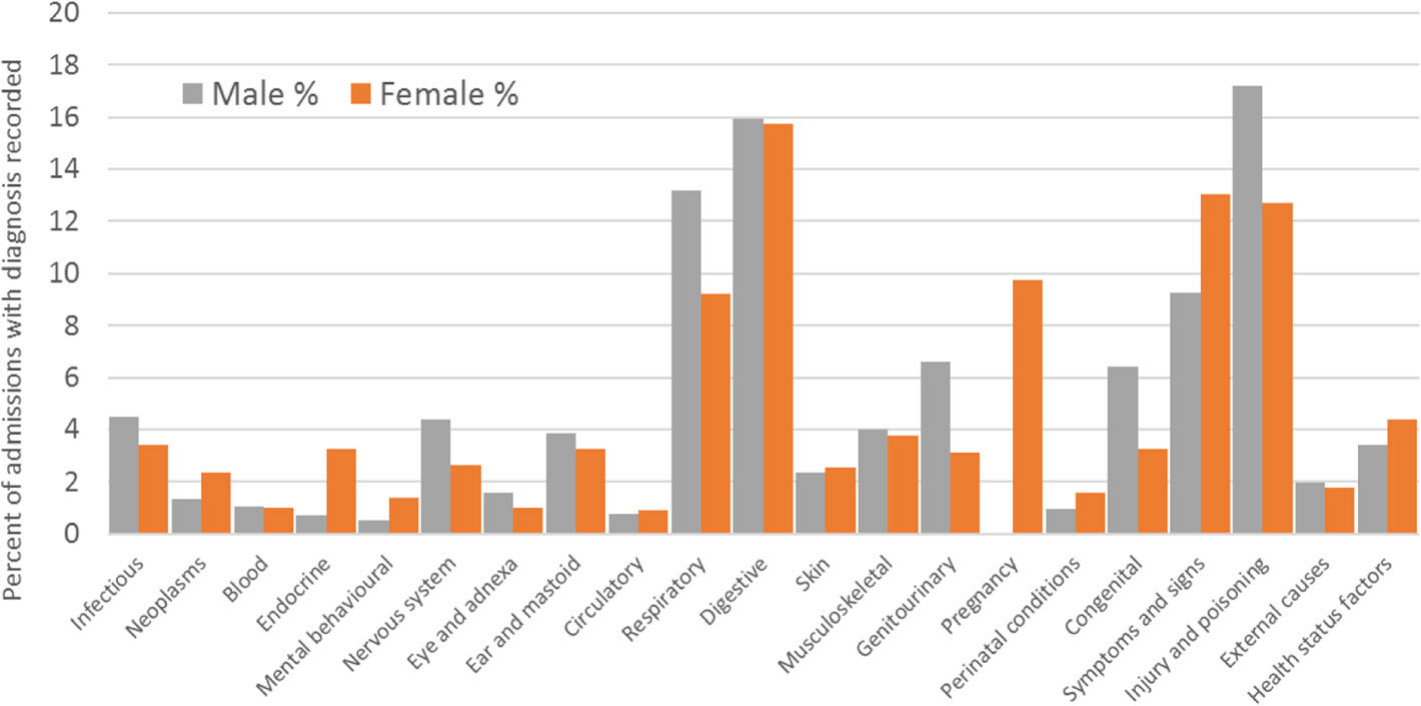
Percentage of ICD primary diagnoses by sex male *n* = 741 (1,693 admissions), female *n* = 1,051 (2,611* admissions). *one admission did not have a primary diagnosis.

**Fig. 4:**
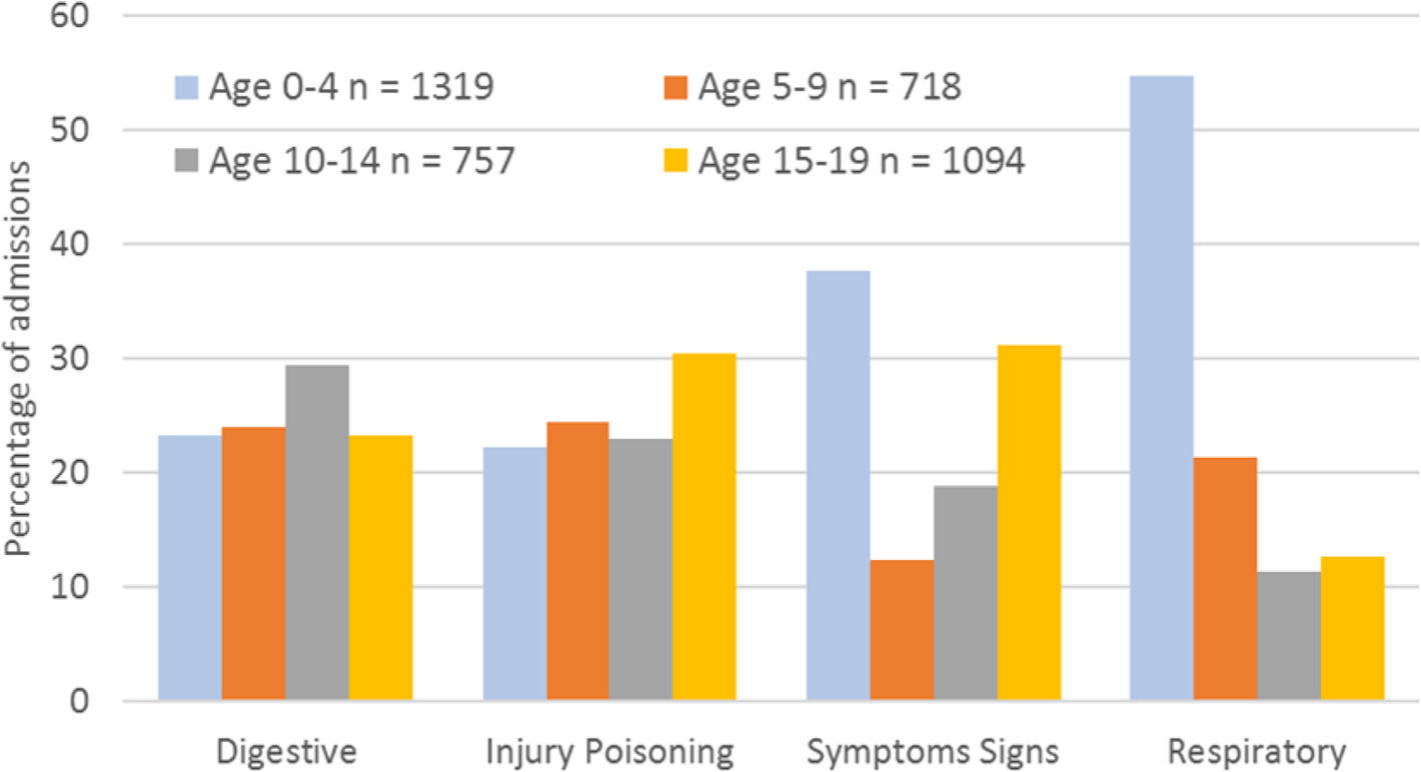
Leading ICD chapters of primary diagnoses by age

The ICD chapter with the highest number of admissions was XI, diseases of the digestive system (ICD10 codes K00-K93), accounting for 681/4,304 (15.8%) of admissions, 270/1,693 (15.9%) among males and 411/2,611 (15.7%) among females (Fig. 3). The chapter with the second highest frequency was XIX, injury, poisoning and certain other consequences of external causes (codes S00-T98) (with 623/4,304 (14.5%) of admissions – 291/1,693 (17.2%) for males and 332/2,611 (12.7%) for females. Males had higher rates of admissions for injury and females had higher rates of admission for poisoning. Within this chapter, 80/623 (12.8%) admissions were for poisoning by drugs, medicaments and biological substances – 8/291 (2.7%) for males and 72/332 (21.7%) for females. Primary diagnoses from chapter XVIII, symptoms, signs and abnormal clinical and laboratory findings, not elsewhere classified (codes R00-R99), accounted for 498/4,304 (11.6%) of admissions – 157/1,693 (9.3%) for males and 341/2,611 (13.1%) for females. The diseases of the respiratory system chapter (chapter X, J00-J99) accounted for 464/4,304 (10.8%) of admissions – 223/1693 (13.2%) for males and 241/2,611 (9.2%) for females. Figure 4 shows the proportion of admissions within these four ICD chapters by 5-year age groups. Respiratory conditions accounted for 54.8% of admissions among those aged 0–4 years.

Tooth decay (Dental caries, unspecified, ICD 10 K02.9) was the highest (four character ICD) primary diagnosis for children aged between 5 and 9 in our cohort, accounting for 46/572 (8.0%) of admissions. Overall, dental caries (ICD 10 K02) accounted for 146/4,304 (3.4%) of all admissions with a primary diagnosis recorded.

The rise in female admissions after the age of 15 (Fig. 2) was partly, but not entirely, due to pregnancy related admissions, which accounted for 254/1,139 (22.3%) of admissions among females aged 15 and over. Primary diagnoses that are part of chapter XVIII (symptoms not elsewhere classified) were also higher for females aged 15 and over, accounting for 164/1,139 (14.4%) admissions compared to 27/373 (7.2%) for males aged 15 and over.

### Emergency readmissions

**Fig. 5:**
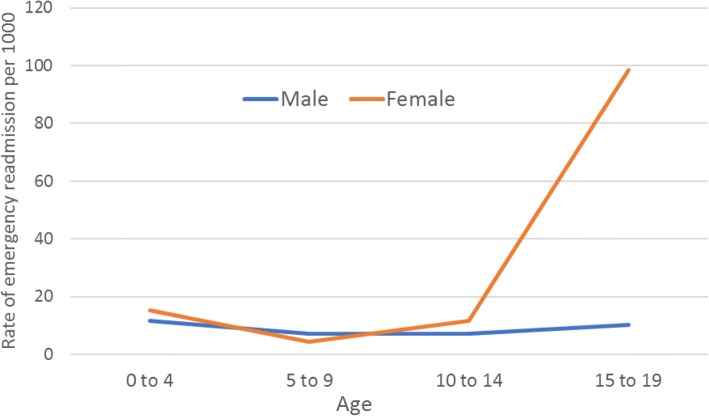
Rate of emergency hospital readmissions by age and sex* (males: *n* = 46 readmissions, 1,281 individuals) (females: *n* = 268 readmissions, 1,908 individuals) *Ages grouped for disclosure control purposes

Of the 4,305 admissions, 314 (7.3%) overall were emergency readmissions. This proportion was higher in females than males (268/2,612 (10.3%) among females compared to 46/1,693 (2.7%) among males). In total, 40 (3.1%) of male participants, and 120 (6.3%) of female participants had at least one emergency readmission (Table 1) and ranged from 0 to 3 readmissions among males and 0 to 31 among females. The rate of emergency readmissions increased markedly by age among females but not males (Fig. 5) – the majority (188/268, 70.1%) of the readmissions among females occurred between the ages 15–19 years and, of these, 83 (44.1%) were pregnancy related. Even after excluding pregnancy-related readmissions, rates of readmission among those aged 15–19 were approximately 5 times higher among females than males. Again, a small number of females accounted for a relatively large proportion of the readmissions: six individuals were readmitted a total of 82 times, accounting for just under one third (30.6%) of the readmissions among females. As with admissions, the pattern remained – but was attenuated – after excluding these six individuals (Additional file 2: Figure S2).

### A&E attendances

**Fig. 6:**
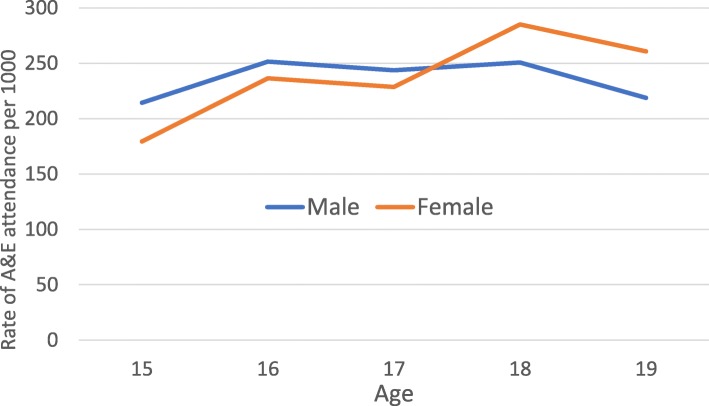
Rates of A&E attendance by age and sex (males: *n* = 1,481 attendances, 1,281 individuals) (females: *n* = 2,132 attendances, 1,908 individuals)

There were 3,613 A&E attendances for the 3,189 participants (mean 1.1, median 0, range 0–93). A greater proportion of males (49.9%) than females (46.1%) had attended A&E at least once (Table 1) and the mean number of attendances was slightly higher among males than females: 1.2 (median 0, range 0–22) vs 1.1 (median 0, range 0–93). As with admissions, there were a small number of females with very high levels of attendance (10 individuals attended A&E a total of 215 times, accounting for 10.1% of the 2,132 attendances among females). Between the ages of 15 and 18, rates of A&E attendance remained relatively stable for males but increased for females (Fig. 6).

### A&E diagnosis codes

**Fig. 7:**
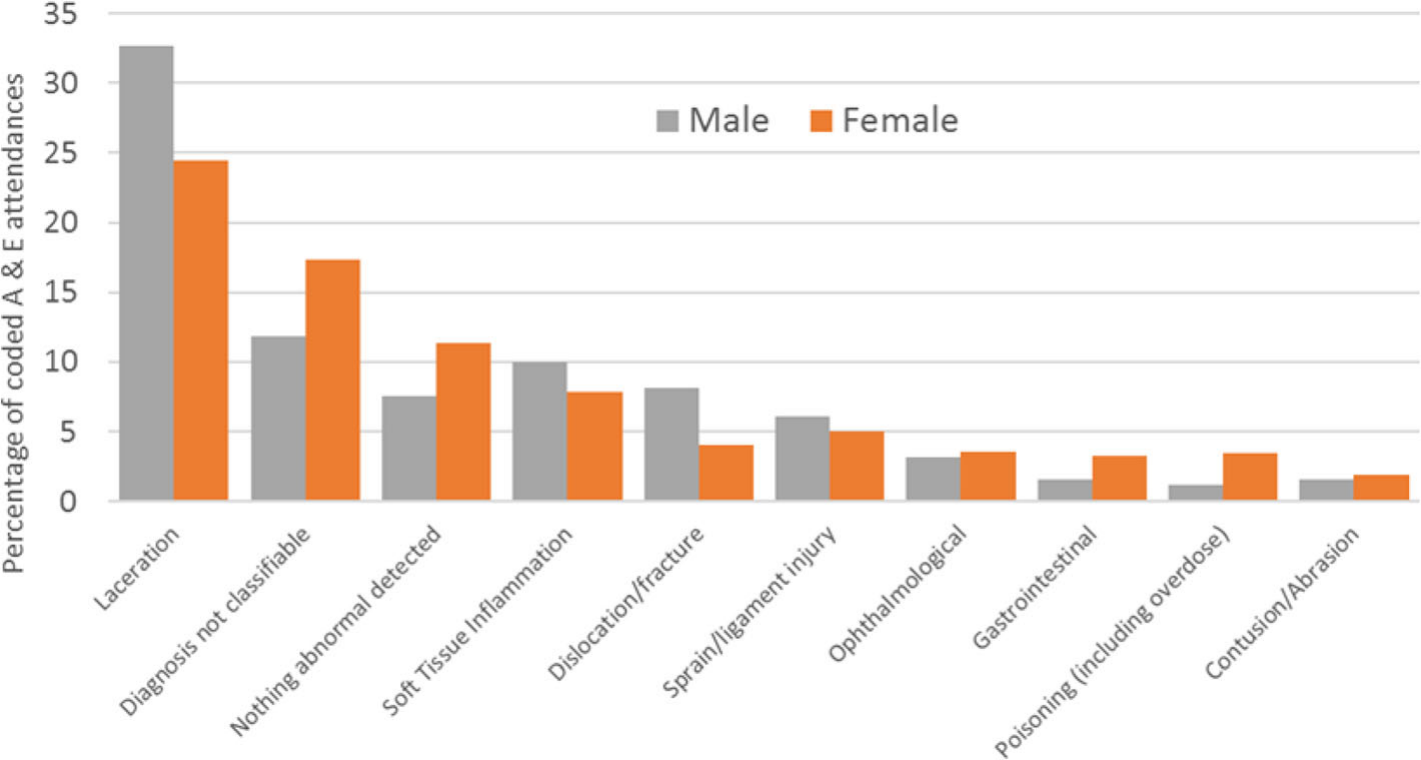
Most frequent A&E diagnoses - percentage of overall A&E attendances with an A&E diagnosis code *n* = 2,513 attendances with an appropriate A&E diagnosis code (male *n* = 1,019, female *n* = 1,494)

For the 3,613 A&E attendances, there were a total of 2,733 (75.6%) recorded A&E diagnoses, thus a quarter were uncoded. 2,513 were given appropriate A&E diagnosis codes, and 220 were given ICD codes in error. The 10 categories with the highest frequency accounted for 2,084/2,513 (82.9%) of all the attendances with a recorded A&E diagnosis code (Fig. 7):

A&E attendances coded as ‘diagnosis not classifiable’, accounted for 121/1,019 (11.9%) of attendances (with a correct A&E diagnosis code) for males, and 259/1,494 (17.3%) of attendances for females. Attendances coded as ‘nothing abnormal detected’ accounted for 77 (7.6%) of attendances for males and 170 (11.4%) of attendances for females.

### Socio-demographic correlates of admissions, emergency readmissions and A&E attendances

These results relate to the 2,774 individuals with complete covariate information. Complete cases were more likely to be female and less likely to be from deprived backgrounds than all enrolled individuals; overall, admission and A&E attendance rates were lower among complete cases compared to all those linked with HES data (Additional file 3: Table S1).

Table 2 gives age and sex-adjusted, plus fully adjusted rate ratios (mutually adjusted for all socio-demographic factors) for admissions, A&E attendances, and emergency readmissions. Most of the socio-demographic factors were strongly associated with admissions in the age and sex adjusted analysis. Admission rates were higher among individuals with lower socio-economic status (as indicated by maternal educational attainment and occupational social class). After mutual adjustment, there were strong associations with occupational social class (higher admissions where parents had manual occupations), maternal educational attainment (higher admissions for lower maternal educational attainment) and maternal age (higher admissions with younger maternal age).

**Table 2:** Socio-demographic correlates of admissions and A&E attendances

	ADMISSIONS		A&E ATTENDANCES		READMISSIONS	
Rates of hospital admission and A&E episode rate	Age and sex adjusted admission rate ratio (95% CI)	Fully adjusted rate ratio (95% CI)	Age and sex adjusted A&E attendance rate ratio (95% CI)	Fully adjusted rate ratio (95% CI)	Age and sex adjusted readmission rate ratio (95% CI)	Fully adjusted rate ratio (95% CI)
Ethnicity (Parent)						
White (Reference)	1.00	1.00	1.00	1.00	1.00	1.00
Other	1.16	1.17	0.22	0.24	0.95	0.49
	(0.77 - 1.76)	(0.78 - 1.77)	(0.10 - 0.33)	(0.14 - 0.40)	(0.15 - 6.27)	(0.06 - 4.04)
	*p* = 0.48	*p* = 0.45	*p* < 0.001	*p* < 0.001	*p* = 0.96	*p* = 0.51
Marital Status						
Married (Reference)	1.00	1.00	1.00	1.00	1.00	1.00
Not Married	1.31	1.11	1.42	1.17	1.49	1.26
	(1.12 - 1.54)	(0.94 - 1.31)	(1.22 - 1.66)	(1.00 - 1.37)	(0.70 - 3.20)	(0.55 - 2.91)
	*p* = 0.001	*p* = 0.21	p < 0.001	*p* = 0.05	*p* = 0.30	*p* = 0.59
Mothers highest education						
Degree / A Level (Reference)	1.00	1.00	1.00	1.00	1.00	1.00
O Level / CSE / Vocational	1.42	1.22	1.83	1.58	1.03	0.77
	(1.26 - 1.59)	(1.08 - 1.38)	(1.63 - 2.05)	(1.40 - 1.79)	(0.56 - 1.89)	(0.40 - 1.50)
	*p* < 0.001	*p* = 0.001	*p* < 0.001	*p* < 0.001	*p* = 0.92	*p* = 0.45
Social Class						
Non-manual occupation (Reference)	1.00	1.00	1.00	1.00	1.00	1.00
Manual occupation	1.56	1.28	1.45	1.01	1.76	1.59
	(1.30 -1.86)	(1.06 -1.54)	(1.21 -1.73)	(0.85 -1.22)	(0.75 -4.14)	(0.62 -4.05)
	*p* < 0.001	*p* = 0.01	*p* < 0.001	*p* = 0.89	*p* = 0.20	*p* = 0.33
Maternal Age						
30+ (Reference)	1.00	1.00	1.00	1.00	1.00	1.00
25–29	1.07	1.06	1.10	1.04	1.29	1.32
	(0.95 - 1.22)	(0.93 - 1.21)	(0.97 - 1.24)	(0.92 - 1.18)	(0.68 -2.45)	(0.67 - 2.61)
< 25	1.92	1.68	2.36	1.95	2.44	2.23
	(1.60 - 2.30)	(1.38 - 2.06)	(1.98 - 2.81)	(1.61 - 2.36)	(1.04 - 5.77)	(0.81 - 6.10)
	*p* < 0.001	*p* < 0.001	*p* < 0.001	*p* < 0.001	*p* = 0.12	*p* = 0.29
Parity						
0 (Reference)	1.00	1.00	1.00	1.00	1.00	1.00
1	0.91	0.96	1.05	1.16	0.87	0.96
	(0.80 - 1.03)	(0.84 - 1.09)	(0.92 - 1.19)	(1.02 - 1.31)	(0.45 - 1.67)	(0.48 - 1.92)
2+	1.12	1.19	1.01	1.16	0.83	0.92
	(0.95 - 1.33)	(1.00 - 1.42)	(0.88 - 1.23)	(0.97 - 1.37)	(0.35 - 1.96)	(0.38 - 2.23)
	*p* = 0.05	*p* = 0.05	*p* = 0.76	*p* = 0.06	*p* = 0.87	*p* = 0.98

All socio-demographic factors except parity were associated with A&E attendances when age and sex adjusted. After mutual adjustment, ethnicity (higher A&E attendances for whites), maternal age (higher attendances with lower maternal age), and maternal educational attainment (higher A&E attendances with lower maternal educational attainment) remained strongly associated with A&E attendances.

Emergency readmissions showed a similar pattern to those seen for overall admissions for factors such as marital status, occupational social class, maternal age and parity but the number of readmissions among our complete case cohort (*n* = 210) was relatively low, resulting in wide confidence intervals.

## Discussion

In this study, we have shown that rates of hospital admission remain relatively stable during late childhood and adolescence among males but increase sharply among females, partly due to pregnancy related admissions. We have found higher rates of hospital admission, emergency readmission and A&E attendance, among individuals from more deprived backgrounds using individual level self-reported indicators of SES from ALSPAC data. The most common primary diagnoses were contained in the ICD chapters for injury and poisoning, digestive and respiratory diseases, and ‘signs and symptoms not elsewhere classified’. A quarter of all A&E attendances were coded as either ‘diagnosis not classifiable’ or ‘nothing abnormal detected’.

Many of our results are consistent with previous research findings. We have found that admissions were higher for males in early life but female admissions were higher, and rose markedly, after the age of 14; this rise was partially – but not entirely – due to obstetric admissions. Similar results have been reported for England as a whole [29]. Others have also reported higher rates of A&E attendance [30] and emergency readmissions among females compared to males during mid-late adolescence and early adulthood, with many readmissions among females being pregnancy-related [26]. The NHS Digital definition of emergency readmissions [24] excludes obstetric care since the readmission may be part of the patient’s care plan. However, even after excluding these, readmission rates were still approximately five times higher among 15–19 year old females than males. We found that 15.1% of A&E attendances were coded as ‘diagnosis not classifiable’. This is in line with national all-age A&E data [31]. In contrast, the number recorded as ‘nothing abnormal detected’ was higher than that reported in national figures for all ages [31]. The highest single primary diagnosis for children aged 5–9 in our cohort was dental caries (tooth decay) and, overall, 8% of all hospital admissions in the ALSPAC cohort were for dental treatment. Public Health England also reported recently that tooth decay is the highest cause of admissions for children in England aged between 5 and 9 [32]; this coincides with grave concern raised by the British Dental Association regarding the provision of dental services for young people [33, 34].

The findings of this study also agree with recent work that has utilised neighbourhood deprivation measures to outline steep socio-economic gradients in hospital use among children and young people in England, both overall [35, 36] and for specific conditions such as injury [8] and poisoning [9, 10]. Part of the explanation for this is a greater incidence of particular health problems, as found in the latter studies of injury and poisoning [8–10]. However, other research has suggested that difficulties accessing primary care services – either actual or perceived – may also play a part, as could dissatisfaction with primary care services [37–40], resulting in lower SES individuals opting to present in hospital rather than in primary care settings [40].

ALSPAC is one of only a few birth cohorts in the UK currently able to use linked hospital records. By linking a cohort study to routinely collected hospital data we have been able to use a wide range of individual level socio-demographic factors in our analysis. Many previous studies have used neighbourhood proxy measures such as the Index of Multiple Deprivation. This can result in weakened estimates of associations with health outcomes, resulting from the fact that the deprivation characteristics of individuals do not necessarily agree with the given characteristics of the area in which they live. As such, our study highlights one of the many potential benefits of establishing linkage between routine health datasets (which contain outcomes for individuals regardless of their participation in research studies) and a longitudinal study containing detailed individual level data.

Our study also has some limitations. The ALSPAC participants whose data have been linked to HES are those who responded positively to the postal campaign. We have found responders to be of higher SES than our cohort as whole; as such the hospital admission and A&E attendance rates observed in this study are likely to be lower than they would be if we had linked HES data for the wider cohort. However, we would expect the overall patterns and relationships to socio-demographic characteristics to be broadly similar. Secondly the HES dataset does not capture all hospital activity in England. Private sector hospital activity is not included, so we are not able to quantify the extent to which the lower levels of hospital activity among higher SES participants are due to private sector hospitalisation. As a guide, in 2014, approximately 11% of the UK population had some form of private health insurance^,^ but there was relatively little private healthcare expenditure on children and young people [41].The impact that this could have on our data is therefore limited. Further, use of the private sector does not affect the finding of this and other analyses showing that users of NHS hospitals will be drawn disproportionately from lower SES groups. We have not used patient address history to determine the denominators for this project. Address history is not complete for all ALSPAC participants, so any method to determine how many participants were living in England at any given time would have been subject to some inaccuracy. We have assumed all individuals for who we established linkage to HES were living in England throughout the period covered. Again, we would not expect this to have affected overall patterns.

NHS hospitals are subject to serious sustainability concerns. Furthermore, NHS primary care organisations in deprived areas are struggling disproportionately with resourcing and workload management [42]. Some evidence suggests that primary care services may have a role to play in reducing hospital admissions, particularly with ambulatory care sensitive (ACS) conditions [43, 44]. The coverage of GPs per head is considerably lower in deprived areas [45], and the Carr-Hill formula, the method of funding for primary care organisations, has been shown to inadequately adjust for deprivation [46]. What is more, the replacement for the Carr-Hill formula distributes proportionately lesser funding to practices in deprived areas compared to Carr-Hill [47]. The Minimum Practice Income Guarantee (MPIG), which has provided financial stability for practices in deprived areas, is being phased out between 2014 and 2021. By reducing, rather than increasing, the capacity of primary care organisations in deprived areas to strategically manage the enhanced health needs of their local populations, these funding changes may exacerbate the current crisis of NHS activity and resourcing. Serious concerns have been expressed that patient safety is being compromised by capacity difficulties in NHS hospitals [48, 49]. If funding allocated to GPs in deprived areas is not sufficient to allow for effective primary care management of patients with enhanced health needs, the flow of patients towards NHS hospitals for unplanned emergency care may be expected to increase.

## Conclusions

Our study demonstrates the benefits offered by linkage between a detailed longitudinal study such as ALSPAC and routine health data. We have carried out a descriptive analysis of hospital admissions and A&E attendances among children and adolescents and, using individual level rather than neighbourhood measures, we have provided further evidence of differences in rates of NHS hospital activity according to socio-economic status. This resource has the potential to be used to carry out further in-depth research into the determinants of hospital use and health outcomes among young people in the UK.

## Additional files

Additional file 1: **Figure S1.** Rates of hospital admission by age and sex with and without the six females with the highest number of admissions. (DOCX 58 kb)

Additional file 2: **Figure S2.** Rates of emergency readmissions by age and sex with and without the six females with the highest number of emergency readmissions. (DOCX 31 kb)

Additional file 3: **Table S1.** Distribution of socio-demographic characteristics among all ALSPAC-enrolled individuals, those who consented to health record linkage and complete cases. (DOCX 16 kb)
