# Supplementary material for: Socio-demographic patterns in hospital admissions and accident and emergency attendances among young people using linkage to NHS Hospital Episode Statistics: results from the Avon Longitudinal Study of Parents and Children
Source: BMC Health Serv Res. 2019 Feb 26;19:134. doi: 10.1186/s12913-019-3922-7 (PMC6390368; doi:10.1186/s12913-019-3922-7)

Supplementary Figure 2

Rates of emergency readmissions by age and sex with and without the six females with the highest number of emergency readmissions


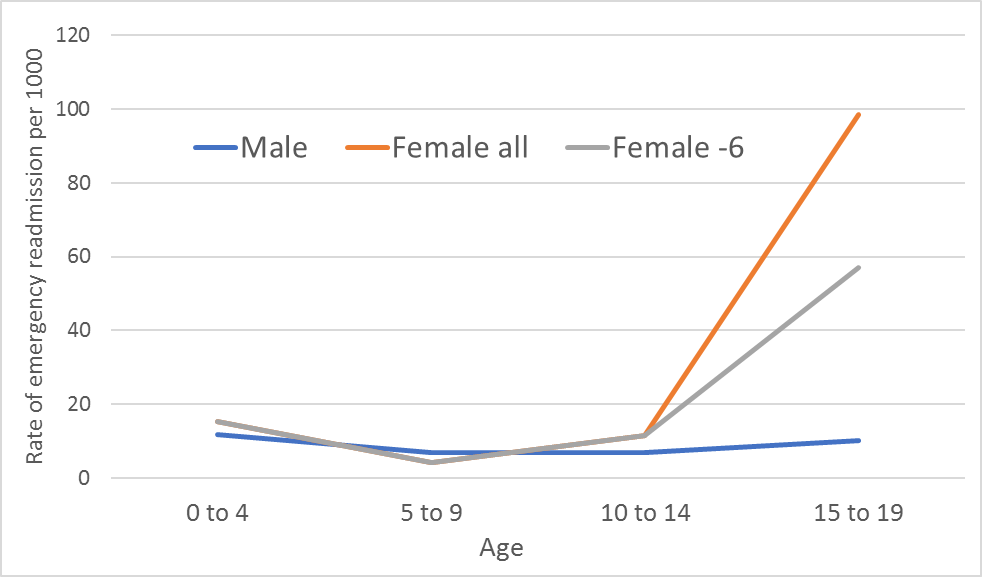

Supplement: Supplementary file 2 — Figure S2. Rates of emergency readmissions by age and sex with and without the six females with the highest number of emergency readmissions. (DOCX 31 kb) [file 12913_2019_3922_MOESM2_ESM.docx]
