# Supplementary material for: Socio-demographic patterns in hospital admissions and accident and emergency attendances among young people using linkage to NHS Hospital Episode Statistics: results from the Avon Longitudinal Study of Parents and Children
Source: BMC Health Serv Res. 2019 Feb 26;19:134. doi: 10.1186/s12913-019-3922-7 (PMC6390368; doi:10.1186/s12913-019-3922-7)
Supplement: Supplementary file 3 — Table S1. Distribution of socio-demographic characteristics among all ALSPAC-enrolled individuals, those who consented to health record linkage and complete cases. (DOCX 16 kb) [file 12913_2019_3922_MOESM3_ESM.docx]

Supplementary Table 1: Distribution of socio-demographic characteristics^1^ among all ALSPAC-enrolled individuals, those who consented to health record linkage and complete cases.

|  | Singletons and twins enrolled in ALSPAC, alive at 1 year and who have not withdrawn from the study (n=14,664)^2^ | All consenters to health record linkage **n = 3189** | Complete cases  **n = 2774** |
| --- | --- | --- | --- |
| Mean APC per participant |  | 1.35 (median 1, range 0-54) | 1.24 (median 1, range 0-24) |
| A&E attendances |  | 1.13 (median 0, range 0-93) | 1.05 (median 0, range 0-30) |
| Emergency readmissions |  | 0.10 (median 0, range 0-31) | 0.08 (median 0, range 0-16) |
| **Sex** |  |  |  |
| Male | 7,523 (51%) | 1,281 (40%) | 1,129 (41%) |
| Female | 7,141 (49%) | 1,908 (60%) | 1,645 (59%) |
| **Ethnicity** |  |  |  |
| White | 12,020 (97%) | 2,984 (98%) | 2,707 (98%) |
| Other | 324 (3%) | 70 (2%) | 60 (2%) |
| **Marital Status** |  |  |  |
| Married (Reference) | 9,789 (75%) | 2,505 (84%) | 2,342 (84%) |
| Not Married | 3,275 (25%) | 496 (17%) | 432 (16%) |
| **Mothers highest education** |  |  |  |
| Degree / A Level (Reference) | 4,385 (35%) | 1,494 (50%) | 1,420 (51%) |
| O Level / CSE / Vocational | 8,009 (65%) | 1,475 (50%) | 1,354 (49%) |
| **Social Class** |  |  |  |
| Non-manual occupation (Reference) | 9,253 (81%) | 2,535 (89%) | 2,471 (89%) |
| Manual Occupation | 2,239 (19%) | 318 (11%) | 303 (11%) |
| **Maternal Age** |  |  |  |
| 30+ (Reference) | 5,224 (37%) | 1,533 (50%) | 1,412 (51%) |
| 25-29 | 5,393 (39%) | 1,118 (37%) | 1,038 (37%) |
| <25 | 3,336 (24%) | 406 (13%) | 324 (12%) |
| **Parity** |  |  |  |
| 0 | 5,765 (45%) | 1,430 (48%) | 1,338 (48%) |
| 1 | 4,530 (35%) | 1,038 (35%) | 986 (36%) |
| 2+ | 2,612 (20%) | 503 (17%) | 450 (16%) |

^1^ Determined during pregnancy

^2^ Denominators vary because the variables come from different questionnaires and not all are complete
